# Transcription clusters and developmental pathways – nature, nurture, noise

**DOI:** 10.1242/jcs.264769

**Published:** 2026-06-18

**Authors:** Peter R. Cook

**Affiliations:** The Sir William Dunn School of Pathology, University of Oxford, Oxford OX1 3RE, UK

**Keywords:** Cell fate, Developmental pathways, Waddington landscape, Transcriptional noise

## Abstract

Establishing how the genomic DNA sequence determines cell fate is a grand challenge in biology. It is usually approached from the viewpoint that each gene is transcribed independently of others. However, there is increasing evidence that clusters of RNA polymerases (variously referred to as transcription factories, condensates and hubs) make most RNA. Here, I use this cluster-based view to present alternative approaches to the grand challenge of linking DNA sequence and cell fate. Artificial intelligence-based tools are driving stunning advances in predicting transcriptional outputs, which in turn direct cell fates; however, they are limited by the curses of dimensionality, data sparsity and understandability. I explore how these AI tools could be used to exploit under-appreciated but information-rich inputs provided by DNA:DNA contacts in clusters.

## Teaser

**Summary:** Noise is everywhere in biology, so how do our tissues develop correctly? This question is addressed from the viewpoint that clusters of RNA polymerases (factories, condensates and hubs) make most RNA.

## Introduction

**Fig. 1.**
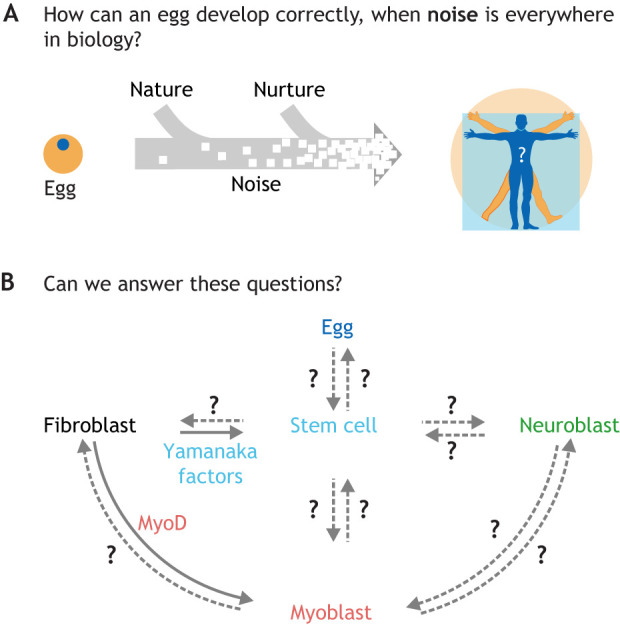
**Overview of a grand challenge.** (A) Tissues in multicellular organisms usually develop in the right places in the right sequence: how do nature and nurture combine to achieve this in the face of inevitable noise? (B) We know how to switch some cell fates, for example, a fibroblast to stem cell switch is achieved by overexpressing Oct4, Sox2, Myc and Klf4 (Yamanaka factors) (Davis et al., 1987), and a fibroblast to muscle myoblast switch is achieved by overexpressing MyoD (Takahashi and Yamanaka, 2016), but it remains to be determined if we can fill in all the question marks.

The transcriptional activity of a gene is ultimately determined by the genomic DNA sequence (‘nature’) with inputs from the surroundings (‘nurture’); understanding how this is achieved is a grand challenge in biology, particularly in the face of inevitable noise due to random fluctuations in the concentration of key molecules within cells (Fig. 1A). Complete understanding could lead to equations that predict the probabilities governing development of an egg into different cell types, or the conditions under which cell fate can be experimentally reprogrammed (Fig. 1B).

Transcription involves assembly of a complex on a chromosome that contains the appropriate promoter, polymerase and factors (Cramer, 2019). I will use the term ‘promoter’ to include a site anywhere in the genome (i.e. both within and outside a gene) that has a high affinity for factors and polymerases that go on to initiate transcription. I also use the term ‘transcription unit’ to include both genic and non-genic sequences. Note that non-genic human promoters and transcription units outnumber genic ones by roughly 10:1, with most of these non-genic sites being enhancers (Andersson et al., 2014).

**Fig. 2.**
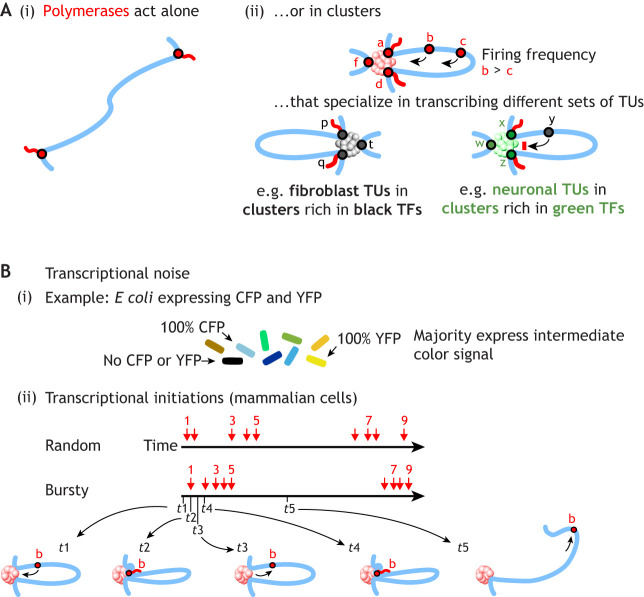
**Models for transcription and transcriptional noise.** Blue lines represent DNA, red circles represent polymerases or transcription factors, and red tails represent nascent RNA. Iconography adapted from Negro et al. (2024). (A) Transcription. (i) Active RNA polymerases are traditionally thought of as acting alone. (ii) An alternative view sees them acting in clusters. Top, active polymerases anchor transcription units (TUs) ‘a’ and ‘d’, transcription factors (TFs) anchor ‘f’, and the firing frequency of any promoter is largely determined by promoter–cluster distance in 3D space (‘b’ diffuses to the cluster and fires more often than ‘c’). Bottom, clusters also specialize in transcribing particular small-world groups of TUs (e.g. black units in black clusters, left), and ‘y’ might visit the green cluster (right), but does not fire there as the cluster lacks appropriate TFs. Clusters are not static, but appear and disappear as TFs, polymerases and DNA bind and dissociate. There are about ten active TUs per cluster in human cells (Negro et al., 2024), and ∼6000 pol II factories in mouse embryonic stem cells (Faro-Trindade and Cook, 2006); TNF, for example, switches on many inflammatory-response TUs in human umbilical vein endothelial cells with 150–250 of them initially being co-transcribed in a (small-world) group of clusters containing the transcription factor NFκB (Papantonis et al., 2012). [For additional quantitative data, see our website: ‘The pan-genomic model: 8 FAQs’ at https://www.petercooklab.uk/pan-genomic-model/8-faqs, accessed 22/04/26.] This means that a unit like ‘p’ is usually co-transcribed with other black units in black clusters, but rarely with ‘q’ and ‘t’ in other cells in the same clonal population. (B) Transcriptional noise. (i) Example. Noisy expression in bacteria of CFP and YFP controlled by the same promoter yields cells expressing many colors (Elowitz et al., 2002). (ii) Types of noise. Nine transcriptional initiations (red arrows) in mammalian gene ‘b’ could occur randomly but usually occur in bursts (two bursts shown in the ‘bursty’ example). Bursting is simply explained by the alternative model as follows. If ‘b’ is tethered close to an appropriate cluster at time *t*1, it is likely to diffuse to (and initiate transcription in) the cluster (at *t*2) before terminating (at *t*3). If still tethered near the cluster, this cycle might repeat (giving initiations 3–5 indicated by the red arrows). If the tether between the DNA and the cluster is lost, *b* might diffuse away (at *t*5) where it has little chance of re-initiating. Consequently, it is silent but might initiate a burst if re-tethered near an appropriate cluster (giving initiations 6–9 indicated by the red arrows). Note that ‘b’ could re-tether near the same cluster, which now contains different TUs, or even near a new cluster that is quite different.

This challenge of linking DNA sequence and cell fate is usually approached from the viewpoint of the traditional model for transcription (Cramer, 2019), where genes scattered around the genome are transcribed independently of others [Fig. 2Ai; for example, see Boyer et al. (2005) and Dekker and Mirny (2016)]. Then, RNA polymerases and the relevant transcription factors diffuse to and bind to appropriate promoters, wherever they happen to be in three-dimensional (3D) space. I approach it from the viewpoint where clusters of RNA polymerases are responsible for most transcription (Fig. 2Aii). These groups of polymerases are referred to as either transcription factories (Cook, 1999; Negro et al., 2024), clusters (Dotson et al., 2022), condensates (Cramer, 2019), drops (Palacio and Taatjes, 2021), pockets (Hilbert et al., 2021) or hubs (Misteli, 2020). I will use the generic term ‘cluster’ for such groups, but conclusions apply generally, as all contain local concentrations of the required machinery that work through the law of mass action to ensure efficient RNA production (e.g. the local concentration of RNA polymerase II in a human factory is ∼1000-fold higher than in the nucleoplasm; Cook, 1999). Then, promoters diffuse through the nucleoplasm, and – if they happen to collide with an appropriate cluster – productive transcription might begin.

There is now increasing evidence for this alternative view (reviewed in Rippe and Papantonis, 2025). Thus, most genomic contacts involve active units. In bacteria, mapping of 3D chromosome conformation by Hi-C shows that active RNA polymerases anchor almost all loops (Bignaud et al., 2024). In mammals, the highest-resolution contact data available shows that 67–74% contacts involve active units (compared to 4% binding CTCF and cohesin, proteins that stabilize many long chromatin loops; Goel et al., 2023). Additionally, there is evidence that almost all transcription occurs in clusters. For example, the human genome encodes hundreds of rRNA genes, but only those clustered in nucleoli are copied by polymerase I (Roussel et al., 1996; Leung et al., 2004). Similarly, >92% of all nascent RNAs made by polymerases II and III are concentrated in extra-nucleolar clusters (Papantonis and Cook, 2013). Related units are also co-transcribed in clusters rich in appropriate transcription factors: gene sets regulated by estrogen receptor (ER)α (also known as ESR1), KLF1, nuclear factor (NF)-κB or TFEC all co-cluster only when active (Fullwood et al., 2009; Schoenfelder et al., 2010; Papantonis et al., 2012; Dotson et al., 2022).

This viewpoint leads naturally to a ‘pan-genomic’ model with two core concepts (Negro et al., 2024) – promoters tethered close to a cluster are more likely to fire than distant ones (in Fig. 2Aii, *b* fires more often than *c*), and different clusters contain different transcription factors that specialize in transcribing related groups of genes (commonly called ‘small-world’ groups; in Fig. 2Aii, black units firing only in fibroblasts are co-transcribed in the black cluster). It also leads naturally to different ways of thinking about how regulatory motifs work and how one might approach the grand challenge of linking sequence to cell fate.

## Noise is inevitable in biology, and biosystems exploit noise

Stochastic fluctuations in signals are usually treated as noise, and signals are lost if noise levels are too high. Intrinsic biological noise stems from random fluctuations in local concentrations of molecules within cells, and extrinsic noise from fluctuations in the surroundings (Elowitz et al., 2002). Perhaps counter-intuitively, complex biosystems often require noise to work (for example, stochastic interactions are integral to appropriate microtubule turnover, chromosome segregation and heart beating (Raj and van Oudenaarden, 2008; Eling et al., 2019, Noble, 2021; Coomer et al., 2022; Meeussen and Lenstra, 2024), and noise can even dilute energy inputs from environmental fluctuations (Shi et al., 2025).

Transcriptional noise refers to the variability in gene expression in genetically identical cells growing under identical conditions (Raj and van Oudenaarden, 2008; Eling et al., 2019), and one of its major causes is ‘bursting’ (Meeussen and Lenstra, 2024). One striking demonstration of such noise involved inserting genes encoding cyan and yellow fluorescent proteins (CFP and YFP) controlled by identical promoters into bacteria; most bacteria expressed intermediate color signal, and some just cyan or yellow (Fig. 2B; Elowitz et al., 2002). Later, fluorescent *in situ* hybridization (FISH) uncovered related variations in CFP and YFP mRNA production, indicating that seemingly identical cells ‘noisily’ produce different levels of RNA and protein (Raj and van Oudenaarden, 2008; Eling et al., 2019).

Bursting arises because promoters do not fire at random but switch between active and inactive states (Fig. 2B; Raj and van Oudenaarden, 2008). Although almost all observed human genes are ‘bursty’, burst frequency is gene specific (Tunnacliffe and Chubb, 2020). Bursts are often described by the rate of switching a burst ‘ON’ and ‘OFF’, plus the initiation rate of polymerase II when ‘ON’ (Meeussen and Lenstra, 2024). However, the conventional model finds it difficult to provide a coherent view of bursting (Lammers et al., 2020; Tunnacliffe and Chubb, 2020). For example, ‘ON’ times are typically minutes to hours (and even days), and this creates variability and noise (Lammers et al., 2020) – but polymerases and transcription factors bind to DNA in seconds. Moreover, different genes behave differently – but polymerases and transcription factors take roughly the same time to diffuse to different promoters. This has been highlighted in a previous Review: “Models with one or two gene states are unable to accurately describe dynamic transcription for many genes” and “Many alternative multistate models have been proposed, but these may be highly context specific” (Tunnacliffe and Chubb, 2020).

In the alternative model, close tethering of a unit to a cluster rich in appropriate factors inevitably ensures frequent visits and so initiation, resulting in a burst (Fig. 2Bii, *t*1–*t*4; Finan and Cook, 2012; Brackley et al., 2021). When close tethering is lost, a unit might diffuse through ‘outer space’ for hours (Fig. 2Bii, *t*5) before it again comes close to an appropriate cluster and reignites another burst (this new cluster could be associated with a different chromosome, which would create a *trans* contact). Note that GFP tagging shows that components of clusters continually exchange with the soluble pool – so that clusters can persist despite replacement over time of all their constituents (Negro et al., 2024).

## Waddington landscapes and noise

The fact that bursting is so poorly understood and that all kinds of noise are so prevalent poses a quandary – how do tissues in multicellular organisms emerge in the right place in the right sequence?

**Fig. 3.**
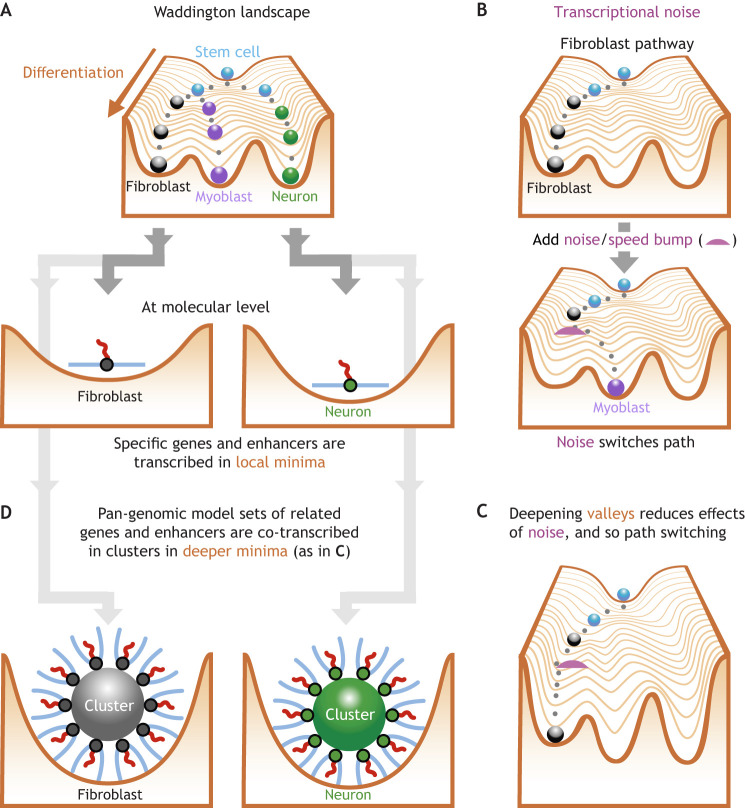
**Waddington landscapes and transcriptional noise.** Iconography adapted from Waddington (1957) and Urban and Johnston (2018). (A) Example landscape. Top, a stem cell (blue ball) differentiates as it rolls down through the landscape towards different valleys leading to different cell fates, presented here as fibroblast, myoblast or neuron. Bottom, according to the traditional model, individual lineage-specific genes are transcribed in different local minima. (B) Transcriptional noise is visualized as a transient ‘speed bump’ that diverts a fibroblast progenitor incorrectly down the myoblast path. (C) Increasing valley depth mitigates the effect of a speed bump. (D) Clusters will be in deeper minima, mitigating effects of transcriptional noise.

Developmental pathways are often visualized as ‘Waddington landscapes’ where ‘hills’ and ‘valleys’ represent ‘free energy potentials’ that guide cells toward the desired state (Fig. 3A, top; Waddington, 1957; Goldberg et al., 2007). According to the traditional model, transcription units transcribed only in fibroblasts or neurons, for example, are found in different local energy potentials that constitute the lowest (and most stable) points in the landscape (Fig. 3A, bottom). Noise is then visualized as a ‘speed bump’ that appears to randomly divert a cell down the wrong path (in Fig. 3B, the bump diverts the progenitor down the unwanted myoblast path; Urban and Johnston, 2018). Given that noise is pervasive, how might its effects be minimized? The obvious way is to ensure that valleys are deeper, so bumps have less of an effect (Fig. 3C).

### Deep minima

Compare transcription of a typical human gene that is either alone (Fig. 3A, bottom) or in a cluster with around nine other units (Fig. 3D). Many factors will influence the equilibrium positions of single active transcription units and clustered ones in the landscape (e.g. concentrations, intermolecular forces, solvent–matrix forces, steric hindrance and landscape shape). Given that we know from theory that entropic forces inevitably drive single units into clusters (Brackley et al., 2013, 2016), and from experiments that >92% nascent RNAs in human and mouse are made in clusters (Papantonis and Cook, 2013), this must mean that the balance of forces ensures that clusters are in deeper free-energy minima than singletons.

Clustering has another consequence – clusters persist for longer than singletons because there are ∼10-fold more DNA-binding sites for relevant polymerases and factors. As global run-on sequencing (GRO-seq) shows the ratio of nascent human mRNAs to eRNAs (enhancer RNAs) is ∼1:10 (Negro et al., 2024), a typical singleton is likely to make one eRNA, compared to a typical cluster polymerizing nine eRNAs plus one mRNA. Making mRNA also takes longer; for example, human RNA polymerase II transcribes a typical eRNA (up to 1 kb) in less than 20 s, and a typical mRNA (of ∼30 kb) in ∼10 min (Jonkers and Lis, 2015). For these reasons, transcribing genes in clusters embedded deep in Waddington landscapes should lessen effects of transcriptional noise.

### Increased transcriptional noise facilitates changes between states

**Fig. 4.**
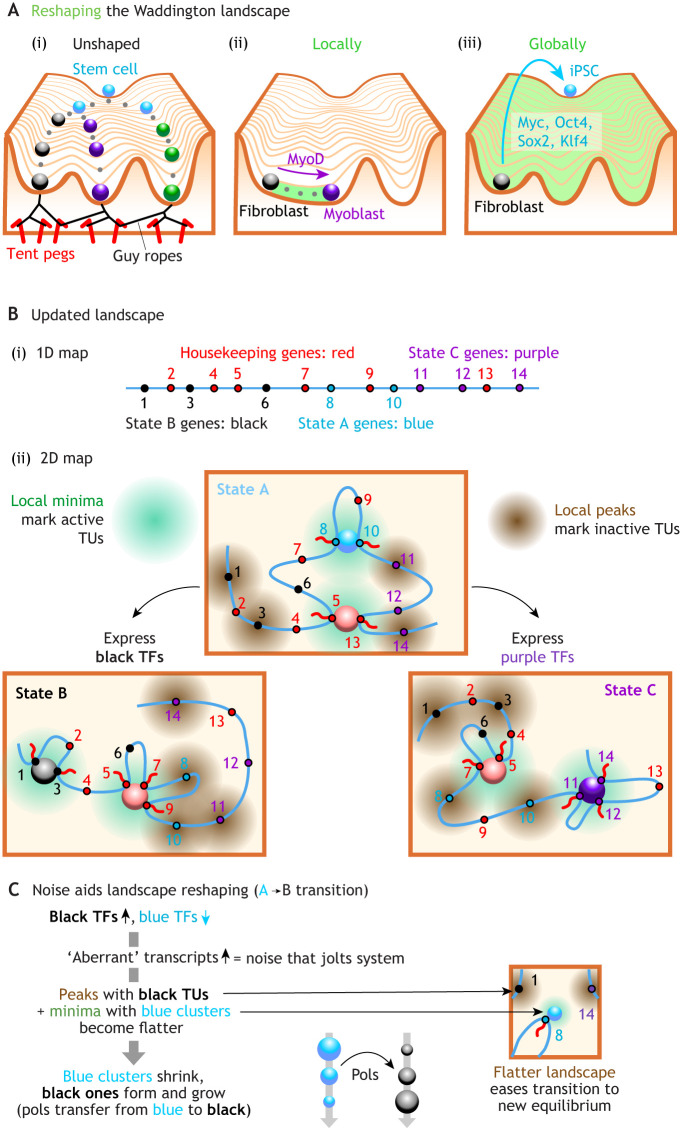
**Noise flattens landscapes at decision points.** (A) Reshaping Waddington landscapes. (i) Waddington imagined his landscape was like the convoluted roof of a tent tethered to the ground through guy ropes. Balls of different colors represent cells progressing towards different cell fates. (ii, iii) Overexpressing MyoD (ii) or the four Yamanaka transcription factors (TFs) Oct4, Sox2, Myc and Klf4 (iii) should locally (or globally) reshape this landscape, respectively. Iconography adapted from Waddington (1957). (B) Updated landscape. Clusters shown as spheres, genome shown as blue lines, and nascent RNAs as red lines. (i) The genetic map indicates units only active in one state (blue, black or purple) plus some active in all states (red). (ii) Some example landscapes. Brown and green represent respectively high and low points in the traditional Waddington landscape (equivalent to inactive and active chromatin, respectively). In state A, red units 5 and 13 plus blue 8 and 10 are transcribed in clusters in local minima (other inactive red units might fire later), and black plus purple units are inactive and at high points (as appropriate TFs are absent). In state B, black and red TFs are present, and black 1 and 3 plus red 5, 7, and 9 are active. In C, red and purple TFs are present, and red 7 and 5 plus purple 11, 12 and 14 are active. (C) As black clusters replace blue ones (clusters shown as spheres), noisy transcription flattens the landscape, making the peaks and minima smaller and easing transition from state A to B. TFs, transcription factors; TUs, transcription units; pol, polymerase.

Waddington imagined his landscape to be like the convoluted roof of a tent viewed from above (Fig. 4Ai; Waddington, 1957). Standing in the tent, one would see many pegs in the ground representing genes and modifying loci (such as expression quantitative trait loci, eQTLs (see below for a definition) and enhancers; GTEx Consortium, 2017; Furlong and Levine, 2018), plus extracellular inputs (from ligands in adjacent cells, growth factors, cytokines, etc.; Hori et al., 2013; Ammeux et al., 2016; Xing et al., 2024). A network of many guy ropes is attached to these pegs, and tension in the ropes integrates inputs to maintain appropriate roof shape. As balls (i.e. cells) pass over the roof, they find themselves at points where they can roll to the left or right, with consequential determination of cell fate. Many factors determine which path is chosen, including stochastic, inductive or selective mechanisms (Till and McCulloch, 1980) plus epigenetic modifications like histone modifications and DNA methylation that stabilize gene expression changes (Vicente-García et al., 2022). I will call all these extracellular inputs ‘nurture’, and one can expect them all to be noisy. Note that grafting experiments in mouse embryos decisively show that these inputs can be strong enough to instruct naïve cells to develop along quite different developmental pathways (Beddington and Robertson, 1989; Beddington, 1994). Strikingly, cells at decision points have long been known to possess a remarkable property – they noisily over-express apparently unwanted transcripts (Chang et al., 2008; Socolovsky et al., 1998; Rosales-Alvarez et al., 2023). This sentiment is captured by this title: “Transcriptome-wide noise controls lineage choice in mammalian progenitor cells” (Chang et al., 2008). Such noise has also been seen to increase when another kind of state changes (i.e. when erythroblasts progress through the mitosis-G1 transition; Hsiung et al., 2016).

Transfection of a cDNA encoding MyoD (herein referring to MyoD1) reprograms human fibroblasts into myoblasts (Davis et al., 1987), and of four cDNAs encoding Oct4 (also known POU5F1), Sox2, Myc and Klf4 convert mouse fibroblasts into induced pluripotent stem cells (iPSCs; Fig. 4Aii and iii; Takahashi and Yamanaka, 2016). From the viewpoint of the conventional model, it is difficult to explain how overexpressing so few protein factors could switch cell fate, when genome-wide association studies (GWAS) point to thousands of non-coding loci scattered around the genome that each have only a tiny effect, but in combination determine phenotypes like those of a fibroblast (GTEx Consortium, 2017). The alternative model again provides a simple explanation; overexpressing a factor >2-fold in simulations simplifies small-world networks, so a master-regulator like MyoD can play a decisive role as the scale of other inputs has shrunk (Brackley et al., 2021). I next propose an updated landscape in which noise plays a critical role in facilitating lineage choice.

In this variant landscape (Fig. 4B), the DNA network organized by clusters performs the integrating function of Waddington's guy ropes by positioning promoters and binding sites of transcription factors in appropriate places in 3D space. When factor concentrations change during development (or by overexpressing MyoD), noise (seen as overproduction of unexpected transcripts) increases (Fig. 4C); then, the network adapts by creating new clusters in new local minima (green hollows). In other words, the system exploits noise to jolt the system and activate transition to a new equilibrium. Thus, assembly of new black clusters in hitherto inactive regions (plus the loss of blue ones in active regions) inevitably reduces heights and depths of pre-existing peaks and hollows (Fig. 4C). This effectively flattens the landscape and brings hitherto inactive black units out of the inactive compartment. As one might expect, the DNA sequence contains the necessary logic encoded in the positions of binding sites for black factors to facilitate the assembly of new black clusters, which grow up to the limit imposed by DNA crowding (Negro et al., 2024). The logic underlying this might appear fuzzy to us, but evolution has honed it to be probabilistically precise (as evidenced by the conserved nature of these sites; Hemberg and Kreiman, 2011; Kim et al., 2025). Of course, movies of 3D volumes containing active units would illustrate such temporal changes better than the 2D maps shown here, but such movies are currently available only from simulations (e.g. Cook and Marenduzzo, 2018).

## *Cis* and *trans* cooperative effects

**Fig. 5.**
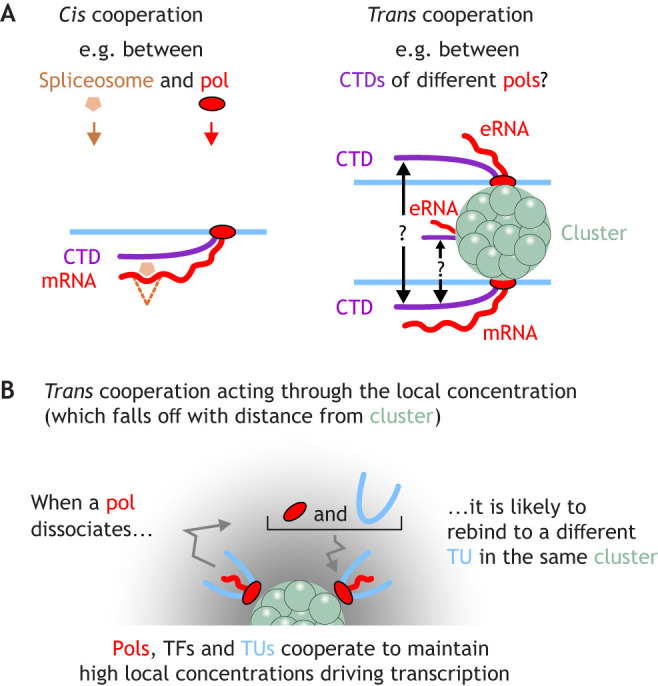
**Cooperative effects in clusters.** (A) *Cis* and *trans* cooperation. Left, the CTD of pol II organizes different machines (here a polymerase and spliceosome). Right, do interactions between CTDs of several polymerases making eRNAs (here two) facilitate mRNA production by another polymerase? (B) Simulations show that *trans* cooperation maintains high local concentrations of the transcription machinery in and around clusters (CTDs not shown), and that clusters are likely to disappear when the local concentration falls below a critical level (Brackley et al., 2013, 2016, 2021). Pol, polymerase; CTD, C-terminal domain; TU, transcription unit; eRNA, enhancer RNA.

Consider the well-established cooperative effects occurring within one polymerizing complex, mediated by the C-terminal domain (CTD) of the largest subunit of polymerase II. I will call such contacts *cis* ones. This CTD can stretch >80 nm away from a polymerase (Cramer et al., 2001) where it coordinates (through physical contact) splicing, poly-adenylation and termination (Fig. 5A; Fong and Bentley, 2001; Jeronimo et al., 2016; Moreno et al., 2023). I suggest several enhancers cooperate through what I will call *trans* contacts by acting on one target gene in a cluster to amplify outputs (Fig. 5A). I suggest we detect such signals as chemical modifications of CTDs, and outputs as increased burst and initiation frequencies.

Another form of *trans* cooperation drives cluster formation and persistence (Negro et al., 2024). As some promoters lie near each other in 3D space and components of the polymerizing machinery bind reversibly, the local concentration of binding sites enhances the chances that dissociated components soon rebind. This also increases the chances that new components diffusing through the locality will be caught in the cluster. This helps cluster growth up to the crowding limit (Fig. 5B). In other words, cooperation of molecules that at any moment are – or were – associated with only one active unit drive clustering of other units, promoting cluster persistence and efficient RNA production.

## Use of AI

A wide variety of datasets are now available to help us address our grand challenge, and we are fortunate that AI models are developing so rapidly and being applied in biology (see examples in Koo and Ploenzke, 2020; Huang et al., 2023; Novakovsky et al., 2023; Sasse et al., 2023; Tang and Koo, 2023; Tang, 2024; Brixi et al., 2026; Consens et al., 2025; Dalla-Torre et al., 2025; Avsec et al., 2026). For example, ‘AlphaGenome’ (Avsec et al., 2026) uses data from 5930 human genome tracks in a 1 Mbp region; these tracks include gene expression and splicing patterns, chromatin states and DNA:DNA contact maps. Although the performance of these AI programs is stunning, fundamental questions remain as to whether they memorize sequence motifs and then regurgitate them, or learn a regulatory grammar and apply its rules to solve new problems (Consens et al., 2025). Moreover, each of the datasets used contains different types and amounts of noise.

The ‘curse of dimensionality’ is a mathematician's phrase (Donoho, 2000) for the intractability of accurately distinguishing signals in noisy datasets like those used by AlphaGenome. Consider one dimension with 10 points on a line (nine being signal, one being noise, and we do not know which is which). In two dimensions there are 10^2^ points, in three 10^3^, and so on – a relentless exponential increase. Consequently, signal becomes sparser and more difficult to detect as evermore datasets are analyzed, and this is accompanied by a relentless decrease in the confidence that signal is being detected rather than noise. Therefore, it seems we should direct attention of AI transformers to the most useful inputs, and I now discuss what these might be, beginning with some used by AlphaGenome.

### The reference sequence of the human genome

The reference sequence of the human genome has more than 3 billion bases, and the number of possible sequences rises as the number of bases, *L*, increases (i.e. *x*∈{A, C, G, T} *L*). Then, with *L*=1, 2, 3, and just 200 bases, the number of possible different sequences rises inexorably from 4, through 16 and 64, to many more than the number of atoms in the known universe (currently ∼10^80^; Tang, 2024). Therefore, the reference sequence is just one of an unthinkably large number of possible sequences (i.e. >10^one billion^). In other words, data such as the reference sequence are truly ‘sparse’ in the context of so many other possibilities – so it is right there are worries that AI platforms will find it difficult to detect signal in the noise (Koo and Ploenzke, 2020; Huang et al., 2023; Sasse et al., 2023; Tang and Koo, 2023; Tang, 2024; Consens et al., 2025). However, there is a glimmer of hope – evolution might have ‘played with’ only a tiny fraction of available sequence space, as the LUCA – the last universal common ancestor of bacteria, archaea and metazoa on our planet (Woese, 2002) – encoded proteins that we recognize today as transcription factors and polymerases (Weiss et al., 2016). Consequently, when evolution chanced upon the first useful sequences in the primordial soup, it stuck with them – and so might have never tested most sequence space. As a result, data in the reference sequence might not be as sparse as superficially expected.

### Transcription factors and their binding sites

There are ∼1600 different genes encoding human transcription factors, with ∼25% being expressed in any tissue (Lambert et al., 2018). Decoding how factors work was recognized to be such a major challenge that the ‘futility theorem’ was applied in the field (Wasserman and Sandelin, 2004; Kim and Wysocka, 2023). This theorem was proposed because there was a three-order magnitude difference between true and false predictions of binding sites – a difference that ensured that essentially all predicted sites had no functional role. I suggest there has been little improvement. Thus, most transcription factor genes encode multiple protein isoforms differing in DNA-binding domains, effector domains or other motifs, and two-thirds of these isoforms have different activities that cannot yet be predicted from sequence (Lambourne et al., 2025). Huge numbers of binding sites are also found in and around promoters (e.g. 2836 and 2472 binding sites are found in 1 kb at the *MYC* and *GAPDH* promoters, respectively (Castro-Mondragon et al., 2022). Most of these binding sites apparently play no functional role (as knocking them out has little effect), most are unoccupied at any moment (Khetan et al., 2025; Mahendrawada et al., 2025), and how close they are to other sites matters (Mahendrawada et al., 2025). Transcription factors are traditionally classified as activators or repressors based on whether they promote or impair transcription, but canonical human activators like NRF1, nuclear transcription factor Y (NFY) and SP1 are now known to repress depending on their position relative to initiation sites (Duttke et al., 2024). Moreover, a systematic survey of all yeast factors has remarkably revealed that many regulatory targets lie far from detectable binding sites (Mahendrawada et al., 2025). We shall see that this is as expected of the alternative model (Negro et al., 2024). Transcription factors were also seen to bind specifically only to DNA, but we now know many also bind to nascent RNA through conserved ‘ARM’ domains (Henninger and Young, 2024). So, it remains to be determined what labels should be attached to these inputs when training our AI transformers.

### eQTLs, enhancers, and silencers

The alternative viewpoint suggests simple mechanisms for the way regulatory motifs work, and our AI models should be told these. Note that the authors of AlphaGenome recognize their focus on a 1 Mbp window misses most inputs from eQTLs and enhancers as they are so widely spread throughout the genome (Avsec et al., 2026). Quantitative trait loci (QTLs) are specific regions of DNA scattered around the genome that influence complex phenotypes like human height and stem-cell fate, and those influencing mRNA levels (and therefore transcription rates) are called eQTLs (GTEx Consortium, 2017). Most eQTLs are single-nucleotide polymorphisms (SNPs) in enhancers and one eQTL or enhancer often targets – and contacts – many genes that are functionally related. Each eQTL has only a marginal positive or negative effect (and so enhances or silences gene activity only slightly), but how eQTLs, enhancers and silencers all work remains unclear (Andersson et al., 2014; Furlong and Levine, 2018; Albert et al., 2018). Note that it is widely agreed that QTLs act post-transcriptionally (as in the omnigenic model for QTL action; Liu et al., 2019), and not co-transcriptionally as imagined here.

In the alternative model, eQTLs, enhancers, plus silencing and boundary elements, are all seen simply as transcription units acting co-transcriptionally, with each being named according to our point of view (Negro et al., 2024). For example, in Fig. 2Aii, unit ‘a’ tethers ‘b’ close to a cluster rich in appropriate factors. This ensures ‘b’ often visits the cluster and so often fires; consequently, we call ‘a’ an enhancer of ‘b’. Similarly, ‘x’ tethers ‘y’ close to an inappropriate cluster (and far from an appropriate one) – and so we call ‘x’ a silencer of ‘y’. With this view, every active genic or non-genic unit can be viewed as simultaneously being one or another type of motif, with firing frequency depending on how closely the motif is tethered to a cluster containing the appropriate factors. Note that eQTLs are uncovered using an unbiased approach, so great weight should be attached to their information. However, they are derived by analyzing levels of steady-state polyadenylated mRNAs and not those of nascent RNAs that are of prime interest here. Therefore, deriving ‘nascent eQTLs’ (neQTLs) using PRO-cap data (derived by sequencing nascent RNAs) would provide an even better input.

### DNA:DNA contacts

Hi-C is currently the most popular way of detecting DNA–DNA contacts, with those stabilized by CTCF and cohesin probably being discussed the most (Dekker et al., 2026). Contacts lie at the core of the alternative model (Fig. 2Aii), but only AlphaGenome among the AI tools cited earlier has used them as inputs (probably because contacts are not central to the traditional model; Fig. 2Ai). I suggest contacts are a precious and under-appreciated ‘super’ input that should be exploited more. Other methods point to contacts beyond those stabilized by CTCF and cohesin as being more numerous and relevant to transcriptional activity. For example, the highest-resolution Hi-C data available (for human lymphoblasts) indicate that the median size of genomic loops anchored by the protein CTCF is ∼360 kbp, but many more loops down to ∼100 kbp are also detected (calculated from the ∼32,000 ‘dots’ seen; Harris et al., 2023). This compares with average loop lengths of <100 kbp determined prior to the introduction of Hi-C (Jackson et al., 1990; Nickerson, 2001). Consequently, this particular Hi-C dataset misses most loops despite containing 42 billion read-pairs from ∼150 experiments. Even so, essentially all active units are found in active regions, termed ‘A’ compartments (as expected of Fig. 2Aii).

In contrast, region-capture micro-C (RCMC) detects short loops better (Gjoni et al., 2025). This high-resolution technique shows that for the *Klf1* and *Ppm1g* loci in mouse embryonic stem cells, 67–74% DNA:DNA contacts involve active promoters and enhancers (as expected in Fig. 2Aii), compared to only 4% for contacts containing CTCF and cohesin (Goel et al., 2023). Note also that the strongest contacts detected by Hi-C are between inactive segments, but those found using genome architecture mapping (GAM) are between active units (Beagrie et al., 2023) – again as expected of the alternative model.

Many Hi-C pipelines also discard three-way and higher-order contacts (Olivares-Chauvet et al., 2016). However, simulations (Brackley et al., 2013) and other techniques such as single-cell split-pool recognition of interactions by tag extension (scSPRITE; Quinodoz et al., 2022), Pore-C (a nanopore-based method; Dotson et al., 2022), and GAM (Beagrie et al., 2023) all yield many higher-order contacts (as expected of the model presented in Fig. 2Aii). Additionally, many Hi-C pipelines exclude *trans* contacts (i.e. ones with other chromosomes). However, scSPRITE gives 54% *trans* contacts compared to just 6% with single-cell Hi-C (Arrastia et al., 2022). Additionally, ‘chromatin interaction analysis with paired-end-tag sequencing’ (‘ChIA-PET’) applied after pulling down polymerase II gives more *trans* contacts than *cis* ones (Li et al., 2012). Similarly, intron seqFISH shows that 82.4% nascent mRNAs in mouse embryonic stem cells lie less than 500 nm from another nascent *trans* mRNA – and so their encoding genes are likely to yield *trans* contacts (Shah et al., 2018).

Taken together, all these results support the idea that clusters are the most important motifs determining structure and function. Therefore, I suggest we should direct our AI models to use contact data (both *cis* and *trans*). Note that AlphaGenome with its 1 Mbp window does not use *trans* information. I also propose we use data from simulations with the fewest possible assumptions, such as those that uncovered why active units spontaneously cluster (e.g. Brackley et al., 2016), data from simple formulae enabling prediction of unit firing frequency (Negro et al., 2024) and data from RNA FISH using probes targeting eRNAs as well as mRNAs, which can also be allied to high-resolution and/or expansion microscopy (Wassie et al., 2019).

## Towards a solution to our grand challenge

As we know from our chatbots, AI tools such as large language models are black boxes, and it is an open question as to whether we will ever know how they derive their output (Colbrook et al., 2022; Campodonico, 2024; Messeri and Crockett, 2024; Zhang et al., 2025). For example, improved robustness of neural networks comes at the cost of reduced accuracy – an ‘uncertainty principle’ that inevitably limits understanding (Zhang et al., 2025). This was captured as an outstanding mathematical problem for the 21st century: “What are the limits of intelligence, both artificial and human?” (Smale, 1998). This prompts me to describe a minimalist and understandable approach that could be used to provide an information-rich input for AI tools.

**Fig. 6.**
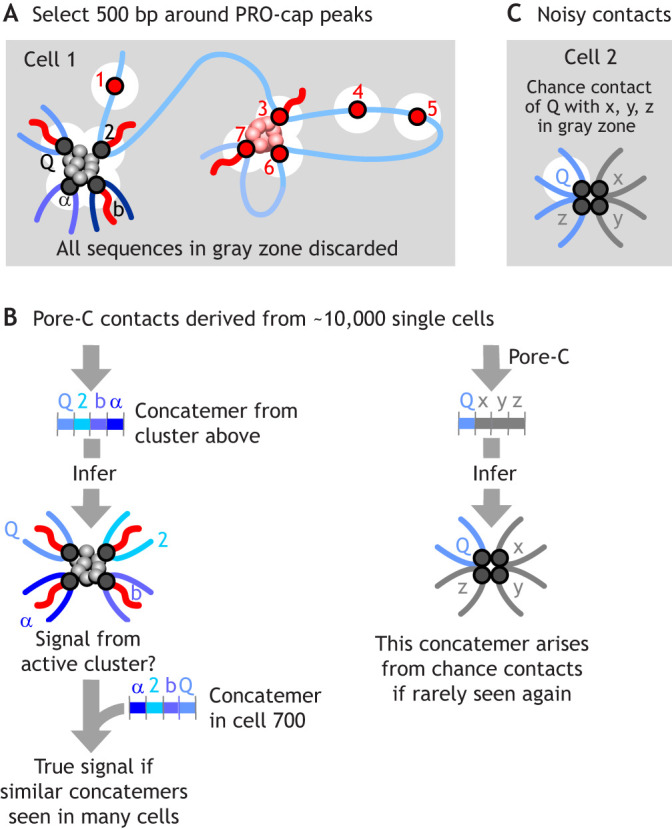
**A proposed minimalist approach for determining chromosome structure and function using just two inputs.** (A) The first input uses population-based PRO-cap data to define all transcription units active in the cell type considered (for example, 500 bp segments around red units 3, 6 and 7 are active in the chromosomal segment shown, but 4 and 5 are currently inactive). (B) The second input uses single-cell Pore-C data (initially only that involving PRO-cap peaks). Here, a Pore-C read yields a concatemer containing peaks found on four different chromosomes (i.e. Q, 2, b and α). The question then arises: are these four segments derived from a cluster like the black one in A? If these segments are found together again in concatemers from other cells, it is likely they were originally co-attached to a cluster. (C) A control allowing one to determine the frequency with which four segments on different chromosomes (i.e. Q, x, y and z) happen to contact each other by chance (such chance events are less likely to recur).

The approach I propose requires two kinds of data. First, sequence space would be reduced by selecting 500 bp ‘windows’ encoding peaks of nascent RNAs around transcription start sites active in the cell type considered (peaks could be obtained using a method like PRO-cap that analyses nascent RNAs; Core et al., 2014). Such ‘windows’ (Fig. 6A, white zones) would contain all sequences involved in determining where most loops are anchored, and where most transcripts are initiated. The rest of the genome in the grey zone (Fig. 6A) would not immediately be considered as it plays a lesser role; however, it will provide non-transcribed ‘control’ sequences. The second input is contact data (both *cis* and *trans*; Fig. 6B), derived using long-read Pore-C and ∼10,000 single cells (or a cell population providing single-allele resolution; Zhong et al., 2023). Contact data is then derived by proximity ligation, with ligation yielding concatemers of sequences that originally lay close to each other in 3D space. Now imagine we see a read derived from a four-way contact involving short ‘segments’ within windows on four different chromosomes (Q, 2, b and α in Fig. 6B). It is possible (although incredibly unlikely) that these four segments result from a chance (noisy) encounter between *Q* and three non-transcribed regions on three chromosomes (as in cell 2 in Fig. 6C). The probability of such chance occurrences can be discovered by analysis of many concatemers like those in cell 2. But if another four-way (or higher-order) concatemer containing segments Q plus 2, b and α (in any order) is seen again in another cell (Fig. 6B, bottom), it becomes highly likely that both Q,2,b,α and α,2,b,Q resulted from pre-existing and functioning clusters (given the numbers and sizes of windows, segments and chromosomes). This follows because certainty that a concatemer is noise-free rises dramatically with increasing numbers of transcription units, concatemers and sightings in different cells (Beagrie et al., 2023). Such concatemers contain various kinds of information, that I now discuss.

First, segments in higher-order concatemers seen many times are likely to bind the same factors that happened to be concentrated in or around clusters of the same ‘color’. Consequently, Q, 2, b plus α are likely to be part of the same (‘black’) small-world network, and 3, 6 and 7 part of a different (‘red’) one (if seen rarely with any one of Q, 2, b, or α; Fig. 6A,B). The size and number of such networks, and how much they overlap, can be determined using standard methods (Koutrouli et al., 2020). Second, which factors cooperate with others could be analyzed using ChIP-seq data (which offers information regarding where different factors bind to DNA) to provide insights relevant to the futility theorem. Third, non-genic units will often be seen together with genic ones, and these should define enhancer and eQTL interactomes. Fourth, the number of times, *n*, a transcription unit is seen in such concatemers should be directly related to firing frequency, *f* (this would be easily confirmed using PRO-cap data). This relationship exists because binding to a cluster is an excellent surrogate for activity, as binding must precede firing when >92% nascent RNAs are made in clusters (Negro et al., 2024). Fifth, results from this proposed approach should validate (or disprove) the model in Fig. 2Aii. Thus, if all units are transcribed in clusters, segments from essentially all windows in the genome should be seen repeatedly in higher order concatemers with other segments that are also seen repeatedly.

This approach is based on the idea that factor concentrations plus the genomic sequence contain all the information needed to form the 3D network of clusters that define a cell state, and that transition to a new state involves increasing noise to jolt the system, flatten the landscape, and lower the activation energy needed to reach a new state. Application of this approach to sets of 10,000 cells of many different types should enable the creation of sets of alternative landscapes, and – with luck – could even enable prediction of which factors to express to switch fate and fill in all the question marks in Fig. 1B.

## Concluding remarks

I have addressed the grand challenge of how our DNA sequence determines cell fate from the viewpoint of an alternative model for transcription where individual polymerases do not act alone, but cluster into transcription factories, hubs or condensates (Fig. 2Aii). I also discussed ways AI models are facilitating stunning advances in predicting outcomes, despite the curse of dimensionality. I contrast this with a minimalist approach (based on the alternative model) that uses just two information-rich sources – PRO-cap data for a cell population, plus long-read Pore-C data from perhaps 10,000 single cells per cell type (Fig. 6).

If we are to solve this grand challenge, we implicitly assume the solution lies within the limits of human intelligence. As for the three-body problem in classical mechanics, there might be no closed-form solution to how three inputs – nature, nurture and noise – interact to determine the path from an egg to a neuron – and so we will have to resort to numerical methods. If we use AI, we must hope the solution lies within the limits of its ‘intelligence’ and that the output can be made understandable to us. As we do not yet know what these limits are, this challenge should prove useful in testing those limits (as recognized by Rajapakse and Smale, 2017). If we use a minimalist and more understandable approach, it might be defeated by data sparsity. Of course, the optimal way is to adopt multiple approaches.
